# Identifying Differentially Expressed tRNA-Derived Small Fragments as a Biomarker for the Progression and Metastasis of Colorectal Cancer

**DOI:** 10.1155/2022/2646173

**Published:** 2022-01-06

**Authors:** Hui Chen, Zhiying Xu, Hua Cai, Ya Peng, Li Yang, Zhen Wang

**Affiliations:** ^1^Department of Gastroenterology, Hunan Provincial People's Hospital, The First-Affiliated Hospital of Hunan Normal University, Changsha, Hunan 410005, China; ^2^Internal Medicine Ward, Hunan Provincial People's Hospital, The First-Affiliated Hospital of Hunan Normal University, Changsha, Hunan 410005, China; ^3^Department of Metabolism and Endocrinology, The Second Xiangya Hospital of Central South University, Key Laboratory of Diabetes Immunology (Central South University), Ministry of Education, National Clinical Research Center for Metabolic Diseases, Changsha, Hunan 410011, China

## Abstract

**Objectives:**

The epithelial-to-mesenchymal transition (EMT) is one key step for the invasion and metastasis of colorectal cancer (CRC). Up until now, the underlying mechanism of EMT in CRC is still unpromising. Thus, it is essential to have a better understanding of its carcinogenesis. The transfer RNA-derived small fragments (tsRNAs) are a new group of small noncoding RNAs (sncRNAs), including tRNA-derived stress-induced RNAs (tiRNAs) and tRNA-derived fragments (tRFs), which have been observed to play an important role in many cancers. However, the relationship between tRFs and EMT in CRC is still unknown. Herein, we aimed to investigate the involvement of tRFs in EMT and its contribution to CRC development.

**Methods:**

We identified the differentially expressed tsRNAs in colorectal cancer cell line HT29 treated with TGF-*β* compared with control cells by using high-throughput sequencing and quantitative real-time reverse transcription-polymerase chain reaction (qRT-PCR). QRT-PCR was conducted to validate the differentially expressed fragments in 68 CRC tumor samples (22 women and 46 men) and adjacent nontumor samples. The association of the expression of tRFs with CRC metastasis and clinical stage was analyzed. Meanwhile, the correlation between tRF expression and overall survival (OS) was also analyzed. TargetScan and miRanda and multiple bioinformatic approaches were used to predict the possible target genes of tsRNAs and analyze possible functions of the tRFs.

**Results:**

A series of differentially expressed tsRNAs were identified in TGF-*β*-treated HT29 cells compared with control cells. tRF-phe-GAA-031 and tRF-VAL-TCA-002 were found to be significantly upregulated in CRC tissues compared to adjacent nontumor tissues. They were significantly correlated with distant metastasis and clinical stage. We compared the differences between tumor samples and nontumor tissues from the ROC curves. The area under the ROC curve (AUC) was up to 0.7554 (95% confidence interval: 0.6739 to 0.8369, *p* < 0.0001) for tRF-Phe-GAA-031 and up to 0.7313 (95% confidence interval: 0.6474 to 0.8151, *p* < 0.0001) for tRF-VAL-TCA-002. For OS analysis, higher tRF-phe-GAA-031 and tRF-VAL-TCA-002 expressions were associated with shorter survival for CRC patients.

**Conclusion:**

A series of differentially expressed tsRNAs are identified in the EMT process of CRC. And tRF-phe-GAA-031 and tRF-VAL-TCA-002 are higher expressed in CRC tissues, and they might play an important role in the metastasis of CRC. Meanwhile, they may be potential biomarkers and intervention targets in the clinical treatment of CRC.

## 1. Introduction

The development of high-throughput RNA sequencing technologies has allowed us to progressively discover thousands of small noncoding RNA (sncRNA), including endogenous siRNAs (endo siRNAs), microRNAs (miRNAs), piwi-interacting RNAs (piRNAs), ribosomal RNAs (rRNAs), transfer RNAs (tRNAs), and transfer RNA-derived small fragments (tsRNAs) [1, 2]. The tsRNAs are derived from the mature or primary tRNAs. Two subtypes of tsRNAs have been reported: tRNA-derived stress-induced RNA (tiRNA) and tRNA-derived fragment (tRF) [3–5]. tiRNAs are generated by a specific cleavage in the middle of mature tRNAs. They can be classified into tiRNA-5 and tiRNA-3 [5]. tRFs are generated through precise processing of both mature and precursor tRNAs (pre-tRNAs) and then classified into four types including the tRF-5, tRF-3, tRF-1, and tRF-2 series [3]. Recently, accumulating evidence has revealed remarkable functions of tsRNAs, tiRNAs, and tRFs, in many physiological and pathological processes, such as protein synthesis, ribosome biogenesis, and cancer transcriptome [4–8].

Recently, a growing number of studies have shown tRFs as novel regulatory molecules implicated in the pathogenesis of cancers [9]. Lee et al. have reported that one tRF-1, namely, tRF-1001, derived from pre-tRNA^Ser^, is highly expressed in several cancer cell lines and is required for proliferation of prostate cancer cells [10]; another tRF, the tRF-3^Thr^, has been demonstrated to be significantly downregulated in primary breast cancer and metastatic tumors, and overexpression of tRF-3^Thr^ in breast cancer cells remarkably inhibited cell invasiveness and migration [11]. tRF-3019a was upregulated in gastric cancer (GC) tissues and cell lines, and tRF-3019a overexpression enhanced GC cell proliferation, migration, and invasion [12]. tRF-Leu-CAG promoted cell proliferation and cell cycle in non-small-cell lung cancer [13]. tRF-03357 promoted cell proliferation, migration, and invasion in high-grade serous ovarian cancer [14].

Colorectal cancer (CRC) is one most common malignance and is one leading cause of cancer-related deaths worldwide [15]. Despite the development of treatments such as surgery and chemotherapy, the prognosis of this disease is still unpromising in many patients due to the presence with metastasis at the time of diagnosis or distant recurrence after therapy [16]. Therefore, it is important to understand the mechanisms by which CRC cells become metastatic. One key step for CRC metastasis is the epithelial-to-mesenchymal transition (EMT), during which epithelial tumor cells lose their polarized organization and cell-cell junctions [17–19]. Intricate positive and negative regulatory processes can regulate EMT. Generally, oncogenic signaling pathways can induce EMT, while tumor-suppressive genes inhibit it. EMT can be induced by a large variety of stimuli, such as transforming growth factor-*β* (TGF-*β*). TGF-*β* belongs to the TGF superfamily, which has been demonstrated to regulate many biological processes, including cell survival, differentiation, and apoptosis [20, 21]. TGF-*β* is also one most important inducer of EMT process. Previous studies have shown that CRC cells presented an enhanced migration capacity after being directly treated with TGF-*β* which might derive from the effect of EMT, since EMT is an important biological process induced by TGF-*β* [20, 21]. Meanwhile, Numbers of miRNAs, lncRNAs, and other types of ncRNAs have been reported to be implicated in the EMT regulatory networks [22–28].

In this study, we aimed to identify dysregulated tsRNAs in TGF-*β*-induced EMT process in CRC cell line HT29, which might provide new perspectives on the mechanisms of EMT, and probably help to better understand the pathogenesis of CRC. We also demonstrated that tRF-phe-GAA-031 and tRF-VAL-TCA-002 were significantly upregulated in CRC tissues when compared with adjacent nontumor tissues. And tRF-phe-GAA-031 and tRF-VAL-TCA-002 were significantly correlated with distant metastasis and clinical stage. Higher levels of tRF-phe-GAA-031 and tRF-VAL-TCA-002 expression were associated with shorter survival. Thus, tRF-phe-GAA-031 and tRF-VAL-TCA-002 may be potential biomarkers and intervention targets in the clinical treatment of CRC. Furthermore, we also used bioinformatic tools to predict possible target genes of the two tRFs and discussed the regulatory mechanisms of them in EMT of CRC. This study will be helpful to screen out potential biomarkers for diagnosis or therapeutic target for CRC.

## 2. Materials and Methods

### 2.1. Tumor Samples

68 CRC tissue samples and matched adjacent normal tissues were obtained from patients who had undergone surgical resection in the Second Xiangya Hospital of Central South University (Changsha, China), between January 2015 and December 2018. Tumor samples were diagnosed according to the World Health Organization (WHO) system, by two pathologists who were unaware of patient data. No radiotherapy or chemotherapy was administered before surgery. The studies involving human participants were reviewed and approved by the ethics committee of the Second Xiangya Hospital of Central South University. All the patients in this study provided their written informed consent.

### 2.2. Cell Culture and Reagents

The human CRC cell line HT29 was cultured in RPMI 1640 medium supplemented with 10% fetal calf serum (FBS) at 37°C with 5% CO_2_. To induce EMT, HT29 cells were seeded into six-well plates at 25% confluence and maintained in a standard medium for 18 h. Then, the cells were starved in 0.5% FBS for 8 h. After starvation, cells were stimulated with TGF-*β* (10 ng/mL, R&D, Minneapolis, MN) in a 0.5% FBS medium for another 48 h to establish the cellular model of EMT.

### 2.3. RNA Isolation and Quantitative Real-Time PCR for the mRNA Expression

Total RNA was extracted from cells by using Trizol reagent (Life Technologies, USA) following the manufacturer's instructions. A total of 1 *μ*g RNA was reverse transcribed to cDNA by using a reverse transcription kit (Fermentas, Glen Burnie, MD, USA). The mRNA expression was assessed using SYBR Premix Ex Taq (Takara, Dalian, China). And GAPDH was used as an internal control. The quantitative real-time PCR assays were performed on the Roche Detection System (Roche Applied Science). The mRNA expression was quantified by a comparative threshold cycle (CT) method and then converted to fold changes. All the experiments were performed at least for three times. The primer sequences used for mRNA expression detection are as follows: Vimentin-F AGATGGCCCTTGACATTGAG, Vimentin-R TGGAAGAGGCAGAGAAATCC; Fibronectin-F GGTGACACTTATGAGCGTCCTAAA, Fibronectin-R AACATGTAACCACCAGTCTCATGTG; MMP-2-F CTGCGGTTTTCTCGAATCCA, MMP-2-R GGGTATCCATCGCCATGCT; ZEB1-F GCACAACCAAGTGCAGAGA, ZEB1-R GCC TGGTTCAGGAGAAGATG; ZEB2-F CAAGAGGCGCAAACAAGCC, ZEB2-R GGTTGGCAATAC CGTCATCC; Slug-F TTCCGATCAGCCTGCCTTTAGA, Slug-R TTTGCCTTGCACAAAGACCAA A; SNAIL-F GCTGCAGGACTCTAATCCAGAGTT, SNAIL-R GACAGAGTCCCAGATGAGCA TTG; CD44-F CTGCCGCTTTGCAGGTGTA, CD44-R CATTGTGGGCAAGGTGCTATT; Twist-F GCCGACGACAGCCTGAGCAACA, Twist-R CGCCACAGCCCGCAGACTTCTT.

### 2.4. RNA Sequence Processing and Data Analysis

The RNA samples were outsourced for library construction and sequenced on the Illumina NextSeq500 System (KangChen Bio-tech, Shanghai, China). Briefly, the RNA samples were pretreated to remove some RNA modifications and were sequentially ligated to 3′ and 5′ small RNA adapters. The RNA was then reversed and amplified. Consequently, ~134-160 bp PCR amplified fragments were purified and used for the preparation of sequencing libraries. And finally, the libraries were sequenced by Illumina deep sequencing. The tRNA sequences of cytoplasmic were downloaded from GtRNAdb, and tRNA sequences of mitochondrial were predicted with tRNA scan-SE software. To generate the mature tRNA libraries, the predicted intronic sequences were moved. Meanwhile, we added an additional 3′-terminal “CCA” to each tRNA. We also included 40 nucleotides of flanking genomic sequence on either side of the original tRNA sequence in order to generate the precursor tRNA libraries. The generated adjusted *p* values lower than 0.05 were considered significant.

### 2.5. Quantitative Real-Time PCR for tRFs

Total RNA extracted from cells and clinical samples were treated with an rtStar™ tRF&tiRNA Pretreatment Kit (Arraystar, USA) to remove RNA modifications that interfere with qRT-PCR assays. Then, the RNA was reversely transcribed by using a Bulge-Loop miRNA qRT-PCR Starter Kit (Ribo, China) according to the manufacturer's protocols. Then, the qPCR assays were performed by using SYBR Green Mix. U6 were used as an internal control. The expression of tRFs was quantified by measuring cycle threshold (Ct) values and normalized using the 2^-*ΔΔ*Ct^ method relative to U6.

### 2.6. Bioinformatic Analyses

The possible target genes of tsRNAs were predicted by miRanda and TargetScan. The tsRNA-mRNA network was then constructed. Gene Ontology (GO) and Kyoto Encyclopedia of Genes and Genomes (KEGG) analysis of these genes were employed for the prediction of biological functions and pathways involved in EMT process of CRC.

### 2.7. Statistical Analysis

Statistical analysis was performed using SPSS17.0 and GraphPad Prism 5. The differences of mRNAs or tRF expression were determined with ANOVA. The associations between dysregulated tRFs and clinicopathological parameters were determined by the chi-square test. The survival curves were estimated by the Kaplan-Meier method. *p* values lower than 0.05 were considered statistically significant.

## 3. Results

### 3.1. TGF-*β* Induces EMT in CRC Cells

**Figure 1:**
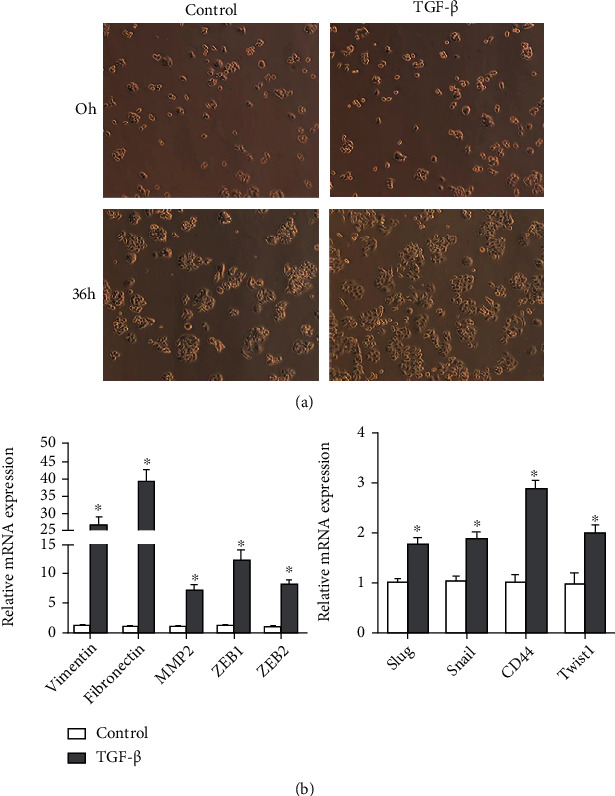
TGF-*β* induces EMT in human colorectal cancer cells. (a) Phase-contrast images of HT29 cells. Human colorectal cancer cell HT29 was starved in 0.5% FBS for 8 h. After starvation, cells were stimulated with TGF-*β* in 0.5% FBS medium for another 48 h to establish the cellular model of EMT. (b) Total RNA was extracted and detected by qRT-PCR for indicated genes in HT29 cells treated with TGF-*β*. All of the genes are involved in EMT. Data are shown as the mean ± SD. *p* < 0.05, compared with control.

To systematically identify tsRNAs that were differentially expressed in EMT process, CRC cell line HT29 was stimulated with TGF-*β* (10 ng/mL) for 48 h to induce EMT. Briefly, HT29 cells were seeded into six-well plates at 25% confluence and grown in a standard medium for 18 h. The cells were starved in 0.5% FBS and then stimulated with TGF-*β* in a 0.5% FBS medium for another 48 h. We observed that TGF-*β* was capable of inducing EMT in HT29 cells, as evidenced by changes in cell morphologies (Figure 1(a)) and changed expression levels of Vimentin, Fibronectin, MMP2, ZEB1, ZEB2, Slug, CD44, Snail, and Twist1 (Figure 1(b)). As expected, TGF-*β* treatment induced an increase in the CRC EMT phenotype.

### 3.2. Differentially Expressed tRFs and tiRNAs in TGF-*β*-Induced EMT Process

**Figure 2:**
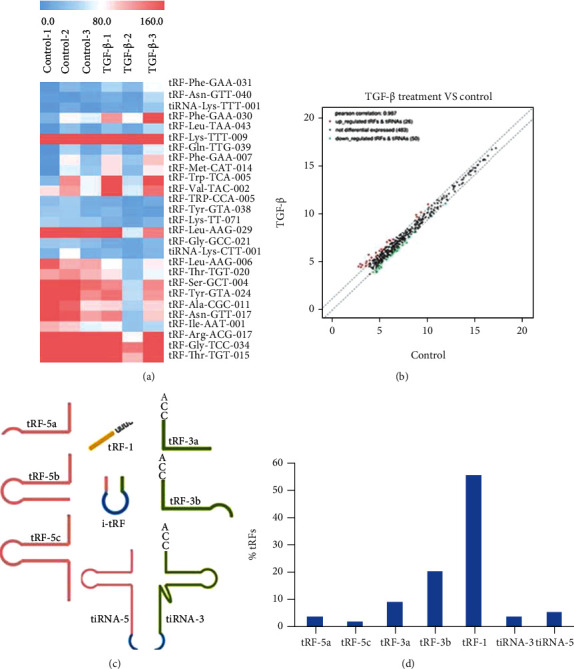
Differentially expressed tRFs in TGF-*β*-treated HT29 cells. (a) The representative differentially expressed tRFs and tiRNAs were compared in a heat map. (b) The scatter plot between the control group and the TGF-*β*-treated cell group. (c) Schematic representation of tRFs and tiRNAs. (d) The contribution of each subtype of differentially expressed tRFs and tiRNAs in TGF-*β*-treated HT29 cells.

We used a high-throughput sequencing technique to screen out differentially expressed tRFs and tiRNAs in TGF-*β*-treated HT29 cells. A total of 559 tRFs and tiRNAs were identified. The distribution of sequence read lengths was recorded (Figure S1), and the copy number of each sample in the distribution of each subtype is shown in Figure S2. Upon further analysis with fold-change filtering (absolute fold change > 1.5), a standard Student's *t*-test (*p* < 0.05), and multiple hypothesis testing (FDR < 0.05), we identified upregulated and downregulated tRFs and tiRNAs in TGF-*β*-treated HT29 cells compared with the control group. The representative differentially expressed tRFs and tiRNAs were compared in a heat map (Figure 2(a)). In addition, the scatter plot indicated the expression level of the two groups (up- and downregulated fragments) between the control group and the TGF-*β* group (Figure 2(b)). Interestingly, it was noticed that 80% of each subtype of differentially expressed tsRNAs were tRFs (Figures 2(c) and 2(d)).

**Figure 3:**
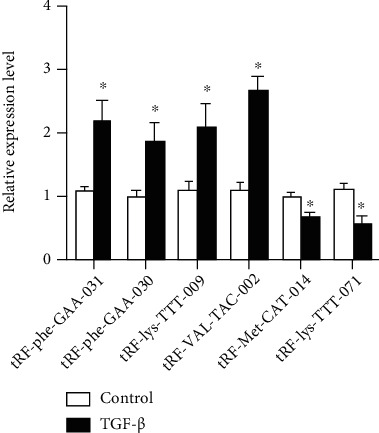
QRT-PCR expression of some tRFs in TGF-*β*-treated HT29 cells and control cells. Gene expression was normalized to U6 expression, and fold differences were calculated using the 2^-△△Ct^ method by comparing gene expression levels to those in HT29 cell. All data were analyzed using the Student's *t*-test. Asterisks indicate significant difference between groups. ^∗^*p* < 0.05.

Moreover, we employed qRT-PCR assays to validate the expression of dysregulated tRFs in TGF-*β*-treated HT29 cells and control cells. The results showed that tRF-Phe-GAA-031, tRF-Phe-GAA-030, tRF-lys-TTT-009, and tRF-VAL-TCA-002 were significantly upregulated in TGF-*β*-treated HT-29 cells, while tRF-Met-CAT-014 and tRF-lys-TTT-071 were downregulated (Figure 3).

### 3.3. Specific tRF Signature Can Serve as Prognostic Marker of CRC

**Figure 4:**
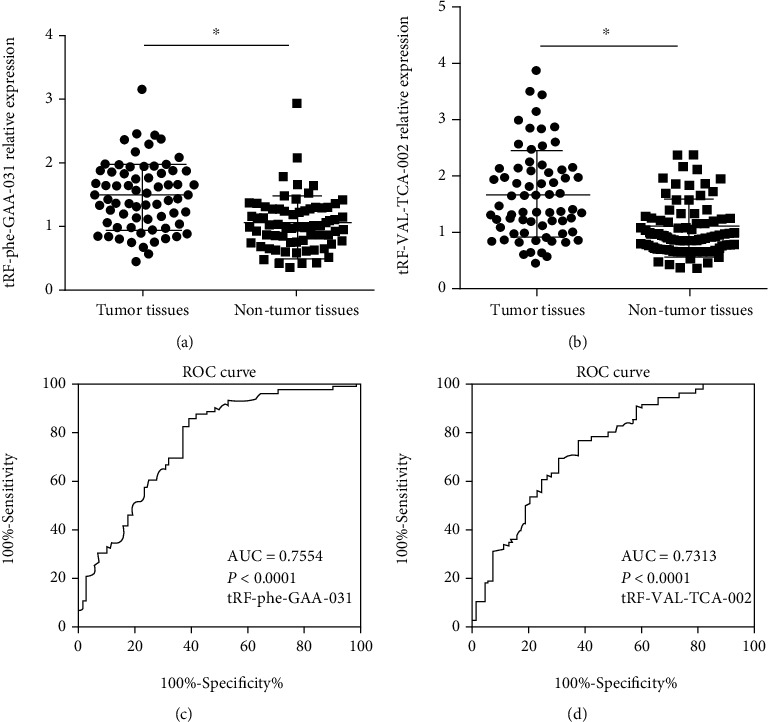
Validation of tRF-phe-GAA-031 and tRF-VAL-TCA-002 expression in tumor tissues and their matched nontumor tissues. (a) tRF-phe-GAA-031 and (b) tRF-VAL-TCA-002 were significantly upregulated in tumor samples. A ROC curve analysis showed the association of (c) tRF-phe-GAA-031 and (d) tRF-VAL-TCA-002 with tumor.

**Figure 5:**
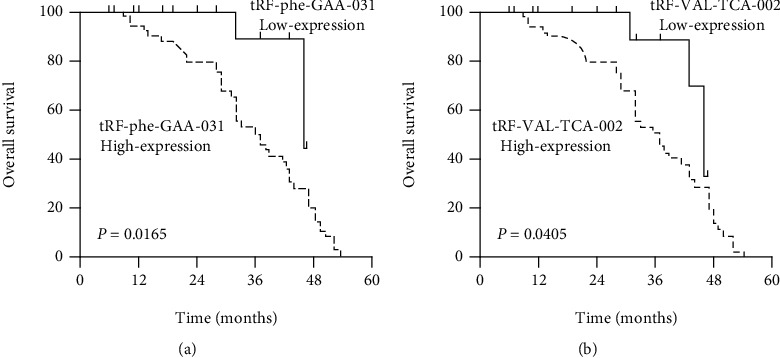
The association of tRFs with OS. (a) Patients with high level of tRF-phe-GAA-031 expression had significantly shorter OS than patients with low level of tRF-phe-GAA-031 expression. (b) Patients with high level of tRF-VAL-TCA-002 expression had significantly shorter OS than those with low expression of tRF-VAL-TCA-002.

In order to determine the relationship between the expression levels of dysregulated tRFs and tumor growth and metastasis, we detected the expression levels of dysregulated tRFs in 68 CRC tumor samples and normal nontumor samples and analyzed the association of dysregulated tRFs with clinicopathologic features. From the data, we observed that the expression of tRF-Phe-GAA-031 and tRF-VAL-TCA-002 was upregulated in tumor tissues compared with their adjacent nontumor tissues (Figures 4(a) and 4(b)). Furthermore, we investigated the associations between the expression level of tRF-Phe-GAA-031 and tRF-VAL-TCA-002 and clinicopathological features of CRC patients. Herein, we defined the expression level of tRF-Phe-GAA-031 > 1.60 as high expression. As shown in Table 1, increased expression of tRF-Phe-GAA-031 was significantly correlated with distant metastasis and clinical stage. Next, we evaluated the diagnostic value of tRF-Phe-GAA-031. We compared the differences between tumor samples and matched adjacent nontumor tissues from the ROC curves. The area under the ROC curve (AUC) was up to 0.7554 (95% confidence interval: 0.6739 to 0.8369, *p* < 0.0001, Figure 4(c)). We also analyzed the expression of tRF-VAL-TCA-002 and found that tRF-VAL-TCA-002 was significantly correlated with tumor differentiation, distant metastasis, and clinical stage (Table 2). The ROC results showed that the area under the ROC curve (AUC) was up to 0.7313 (95% confidence interval: 0.6474 to 0.8151, *p* < 0.0001, Figure 4(d)). In addition, the relationships between tRF-Phe-GAA-031 or tRF-VAL-TCA-002 expression and patient overall survival were also evaluated by Kaplan-Meier analysis. The data indicated that patients with higher tRF-Phe-GAA-031 and tRF-VAL-TCA-002 expression had a poor OS (*p* < 0.001, Figures 5(a) and 5(b)). Collectively, these data revealed that tRF-Phe-GAA-031 and tRF-VAL-TCA-002 might be potential diagnostic and prognostic biomarkers for CRC.

### 3.4. Prediction of Target Genes of the Two tRFs by Bioinformatic Tools and GO and KEGG Analyses

**Figure 6:**
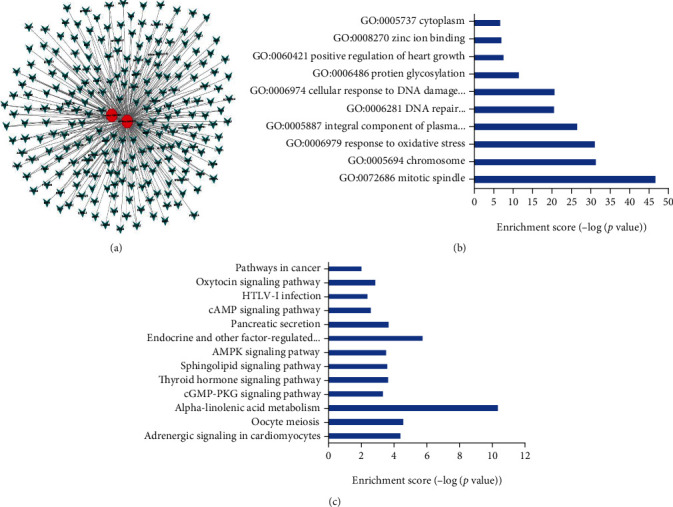
Prediction of target genes of tRFs and GO and KEGG analyses. (a) The network of tRF-phe-GAA-031 and tRF-VAL-TCA-002 and predicted target mRNAs. (b) Top significant GO terms associated with the predicted target genes of tRF-phe-GAA-031 and tRF-VAL-TCA-002. The horizontal axis represents the GO category, and the vertical axis represents the enrichment score. (c) Top significant KEGG pathways associated with the predicted target genes of tRF-phe-GAA-031 and tRF-VAL-TCA-002. The horizontal axis represents the GO category, and the vertical axis represents the enrichment score.

In accordance with the predicted target genes of tRF-Phe-GAA-031 and tRF-VAL-TCA-002 based on TargetScan and miRanda, we identified 299 target genes associated with the two tRFs. These target genes were selected with significant probability (for miRanda database, energy < −15; for TargetScan database, context + scores < −0.4). The network of tsRNA-mRNA was constructed (Figure 6(a)).

Analyses of the GO and KEGG pathways were performed according to the predicted target genes of tRF-Phe-GAA-031 and tRF-VAL-TCA-002 to further explore the possible involved functions and pathways of the two tRFs. The GO enrichment analysis was conducted mainly on three domains, namely, biological process (BP), cellular component (CC), and molecular function (MF). GO analysis enriched terms such as mitotic spindle, response to oxidative stress, and integral component of plasma membrane. And KEGG analysis enriched pathways including alpha-linolenic acid metabolism, PI3K-Akt signaling pathway, and pathway in cancer (Figures 6(b) and 6(c)).

## 4. Discussion

CRC is one most common cancer in the world. Despite recent improvements in the diagnosis and treatment for the disease, the prognosis is still poor for many patients due to the recurrence and distant metastasis [15]. Therefore, a better understanding of cancer carcinogenesis and in-depth exploration of diagnosis biomarker for CRC is still needed. The tsRNAs are a group of small ncRNAs, which are classified into two groups: tiRNAs and tRFs. With the development of high-throughput RNA sequencing technologies, tsRNAs have been identified to implicate in many human diseases, such as cancer, infection, neurodegeneration, and other pathological conditions [24, 28, 29]. And dysregulated tRFs and tiRNAs in cancers were considered as potential diagnostic biomarkers or therapeutic targets in several types of cancers. However, the roles of tRFs and tiRNAs have not been clearly clarified.

The epithelial-to-mesenchymal transition (EMT) is the key step for CRC metastasis [17–20]. TGF-*β* is one dominant cytokine that can induce EMT process, during which, the epithelial cells will symbolize as decreasing epithelial markers such as E-Cadherin, ZO-1, and increasing mesenchymal markers such as N-Cadherin, Vimentin. In order to discover new regulators involved in EMT process and metastasis, we used a TGF-*β*-induced EMT cellular model and analyzed the change of expression pattern of tRFs and tiRNAs in this process. In the present study, we observed that TGF-*β* treatment could induce EMT process in CRC cell line HT29, as evidenced by changes in cell morphologies and changed expression levels of Vimentin, Fibronectin, and others. This observation is consistent with the previously studied EMT models. To explore differentially expressed tsRNAs in the EMT process of CRC cells, we performed high-throughput sequencing to determine the expression profile of tRFs and tiRNAs in TGF-*β*-treated HT29 cells. Based on the sequencing data, we conducted a comprehensive analysis of tsRNAs in TGF-*β*-treated cells in comparison with control groups. The sequencing data showed a series of dysregulated tRFs and tiRNAs in TGF-*β*-treated HT29 cells, which indicated that tRFs and tiRNAs might be involved in the EMT process of CRC cells. tiRNAs can further be classified into tiRNA-5 and tiRNA-3, while tRFs are classified into four types including the tRF-5, tRF-3, tRF-1, and i-tRF series [2, 10]. To be of interest, in our study, the majority of differentially expressed tsRNAs were tRFs. These tsRNAs and tRFs are obtained from the databases, including GtRNAdb and tRFdb. Interestingly, it was noticed that 80% of each subtype of differentially expressed tsRNAs were tRFs. Meanwhile, we selected 4 upregulated tRFs, including tRF-Phe-GAA-031, tRF-Phe-GAA-030, tRF-lys-TTT-009, and tRF-VAL-TCA-002, and 2 downregulated tRFs, including tRF-Met-CAT-014 and tRF-lys-TTT-071, to validate their expression by qRT-PCR. The tsRFs belong to the tRNA-derived fragment (tRF) family. The results showed that the results are consistent with the sequencing data.

To further confirm the association of dysregulated tRFs with CRC, we determined the expression levels of tRF-phe-GAA-031 and tRF-VAL-TCA-002 in tumor tissues and adjacent nontumor tissues. The results showed that tRF-phe-GAA-031 and tRF-VAL-TCA-002 were upregulated in CRC samples. Additionally, higher tRF-phe-GAA-031 expression was correlated with distant metastasis, and clinical stage, while tRF-VAL-TCA-002 was also related with tumor differentiation as well as distant metastasis, and clinical stage. Meanwhile, we compared the difference of tRF-phe-GAA-031 and tRF-VAL-TCA-002 between CRC tissues and adjacent nontumor tissues from the ROC. The area under the ROC curve (AUC) for tRF-phe-GAA-031 was up to 0.7554 (95% confidence interval: 0.6739 to 0.8369, *p* < 0.0001), while for tRF-VAL-TCA-002, the area was up to 0.7313 (95% confidence interval: 0.6474 to 0.8151, *p* < 0.0001). And the overall survival analysis showed the patients with higher tRF-phe-GAA-031 and tRF-VAL-TCA-002 expression had worse survival. These findings indicated that upregulated tRF-phe-GAA-031 and tRF-VAL-TCA-002 may be involved in the tumorigenesis of CRC, and it could be a good diagnostic and prognostic biomarker for CRC.

Previous studies have shown that tRF/miR-1280 is downregulated in tumor tissues. tRF/miR-1280 overexpression could suppress cell proliferation and colony formation, whereas knockdown of it would partially reverse these effects. Interestingly, tRF/miR-1280 reduces tumor formation and metastasis by directly targeting the Notch ligand JAG2, which is essential for cancer stem-like cells (CSC) in CRC progression [29]. However, the roles and the functional mechanisms of tRF/tiRNA-mediated colorectal cancer EMT are still at its infancy. Different types of tRFs have a variety of different functional mechanisms. 5′-tiRNAs play a role in inhibiting protein synthesis and promoting the assembly of stress granules, whereas tRFs have been identified to copurify with Argonaute and piwi complexes, which reveals that such tRFs could act as miRNAs or piRNAs. Recently, 5′-tRF series were also found to inhibit translation independently of siRNA. Specific i-tRFs have been shown to compete with mRNAs for the binding to RNA-binding proteins [4, 30]. To further explore the possible mechanisms of tRF-phe-GAA-031 and tRF-VAL-TCA-002, the possible tRF-mRNA network was constructed. Moreover, by GO and KEGG analyses, we detected several potential biological functions and possible involved pathways that might contribute to CRC pathogenesis, which also provided potential novel targets for therapies of CRC. However, we have no further research about the specific mechanisms of tRFs in CRC. Thus, the mechanism underlying the effects of tRFs on EMT in CRC extremely needs further investigation.

## 5. Conclusion

In conclusion, our study provides a comprehensive analysis of dysregulated tsRNAs in the EMT process of CRC cells. We also identified tRF-phe-GAA-031 and tRF-VAL-TCA-002 as potential diagnostic and prognostic biomarkers for CRC. And they might be possible diagnostic biomarkers and intervention targets for CRC in the future. We believe that these data will help to clarify the pathogenesis of CRC and could provide enlightening ideas for biological functions of these novel tRFs in CRC.

## Figures and Tables

**Table 1:** Association of tRF-phe-GAA-031 expression with clinical and pathologic features in CRC patients.

Parameter	Total	tRF-phe-GAA-031		*χ* ^2^	*p*
		Low	High		
Age (years)				0.981	0.322
≤50	16	2	14		
>50	52	15	37		
Gender				0.090	0.765
Female	22	6	16		
Male	46	11	35		
Tumor size (cm)				2.375	0.123
<4	33	11	22		
≥4	35	6	29		
Tumor differentiation				0.968	0.325
Well/moderate	37	11	26		
Poor	31	6	25		
Distant metastasis				5.181	0.023
Negative	28	11	17		
Positive	40	6	34		
Clinical stage (TNM)				8.586	0.003
I~II	24	11	13		
III~IV	44	6	38		

**Table 2:** Association of tRF-VAL-TCA-002 expression with clinical and pathologic features in CRC patients.

Parameter	Total	tRF-VAL-TCA-002		*χ* ^2^	*p*
		Low	High		
Age (years)				25	0.114
≤50	16	3	13		
>50	52	15	37		
Gender				0.011	0.917
Female	22	6	16		
Male	46	12	34		
Tumor size (cm)				1.551	0.213
<4	33	11	22		
≥4	35	7	28		
Tumor differentiation				2.175	0.014
Well/moderate	37	12	25		
Poor	31	6	25		
Distant metastasis				6.567	0.01
Negative	28	12	16		
Positive	40	6	34		
Clinical stage (TNM)				10.55	0.001
I~II	24	12	12		
III~IV	44	6	38		

## Data Availability

The original contributions presented in the study are included in the article/Supplementary Material; further inquiries can be directed to Zhen Wang.

## Abbreviations

sncRNA: Small noncoding RNA
endo siRNAs: Endogenous siRNAs
miRNAs: MicroRNAs
piRNAs: Piwi-interacting RNAs
rRNAs: Ribosomal RNAs
tRNAs: Transfer RNAs
tsRNAs: Transfer RNA-derived small fragments
tiRNAs: tRNA-derived stress-induced RNAs
tsRFs: tRNA-derived small RNA fragments
pre-tRNAs: Precursor tRNAs
CRC: Colorectal cancer
EMT: Epithelial-to-mesenchymal transition
OS: Overall survival.

## Supplementary Materials

Supplementary Materials Figure S1: the distribution of sequence read lengths between the control group (NC) and the TGF-*β* treatment group. Figure S2: the copy number of each sample in the distribution of each subtype between the control group (NC) and the TGF-*β* treatment group.
