# Supplementary material for: Identifying Differentially Expressed tRNA-Derived Small Fragments as a Biomarker for the Progression and Metastasis of Colorectal Cancer
Source: Dis Markers. 2022 Jan 6;2022:2646173. doi: 10.1155/2022/2646173 (PMC8758288; doi:10.1155/2022/2646173)
Supplement: Supplementary Materials — Figure S1: the distribution of sequence read lengths between the control group (NC) and the TGF-β treatment group. Figure S2: the copy number of each sample in the distribution of each subtype between the control group (NC) and the TGF-β treatment group. [file 2646173.f1.docx]

Supplementary File:


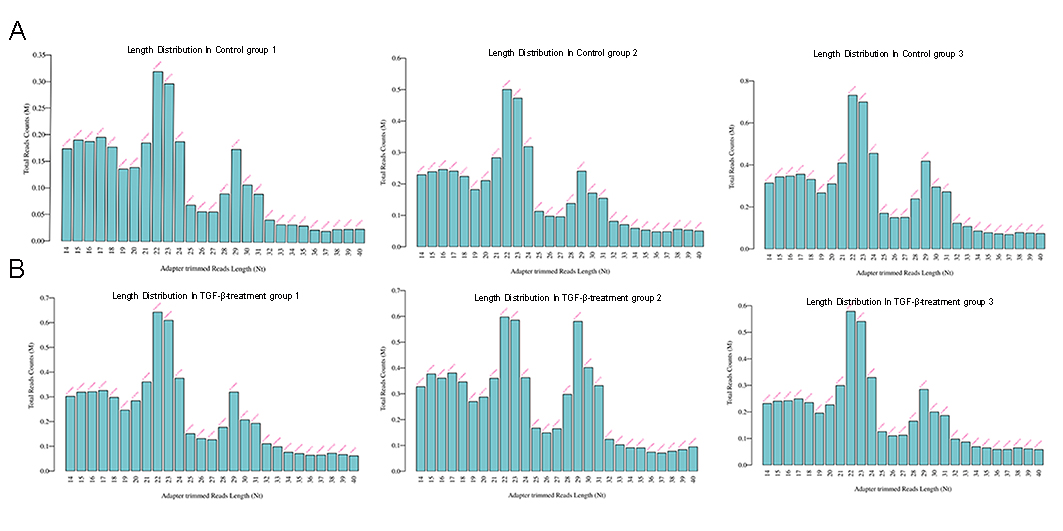


Fig. S1 The distribution of sequence read lengths.


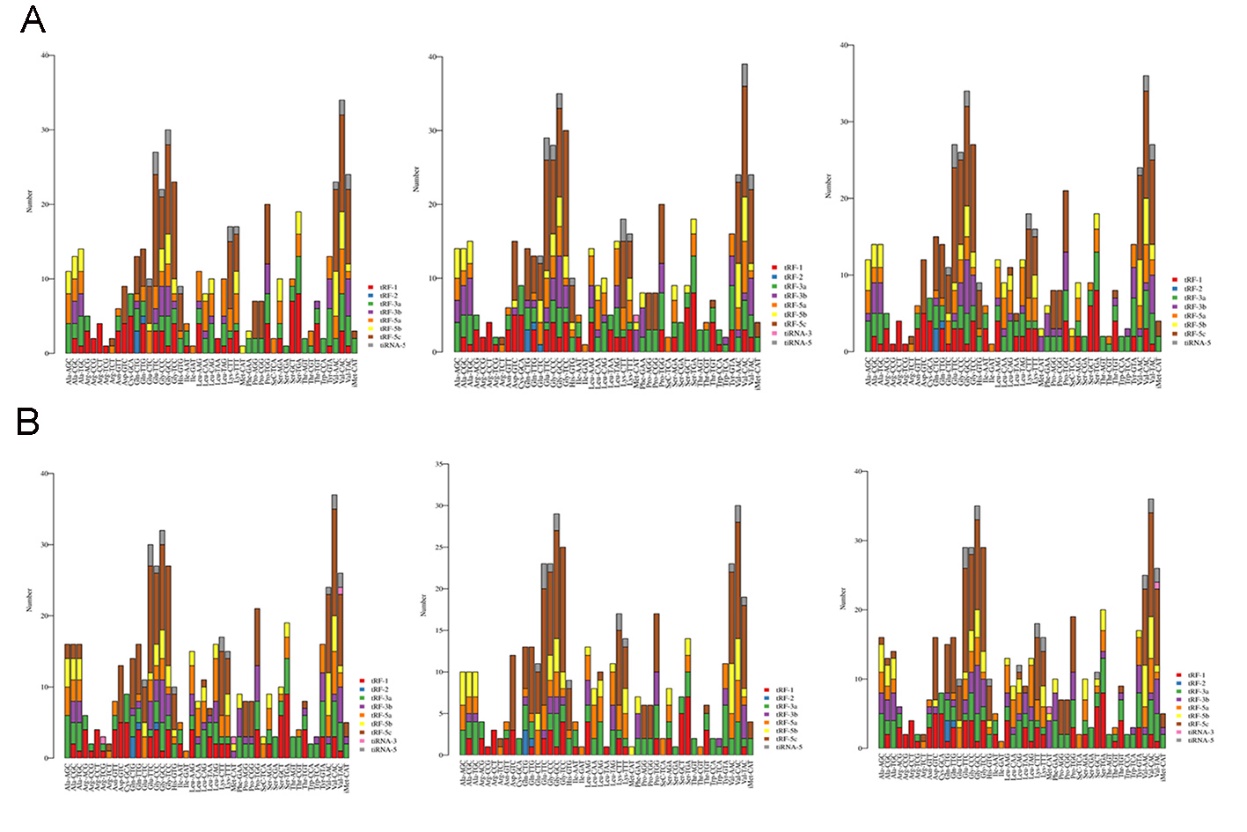


Fig. S2 The copy number of each sample in the distribution of each subtype.
